# Osteoblastoma of Talus: A Diagnostic Dilemma

**DOI:** 10.7759/cureus.11838

**Published:** 2020-12-02

**Authors:** Sarang Agarwal, Dharmendra K Singh, Ashish Rustagi, Loveneesh Krishna, Jatin Talwar

**Affiliations:** 1 Orthopaedics, Vardhman Mahavir Medical College and Safdarjung Hospital, New Delhi, IND; 2 Radiology, Vardhman Mahavir Medical College and Safdarjung Hospital, New Delhi, IND

**Keywords:** osteoblastoma, talus, diagnosis, multidisciplinary approach

## Abstract

The critical biomechanical importance of talus and nonspecific clinical features of talus lesion warrants a meticulous diagnostic work-up for specific management, particularly when the talus lesion is associated with concomitant soft tissue and joint abnormalities. We present a rare case of osteoblastoma of talus with concomitant tenosynovitis of tibialis anterior, ankle joint effusion, varicose vein and moderate distal arterial stenosis.

## Introduction

Bone tumors of the foot comprise of approximately 3% of all osseous tumors. Osseous tumors of talus have been reported to be between 8% to 23% of tumoors in the foot [1]. The common osseous tumors of talus are chondroblastoma, osteoid osteoma, giant cell tumor, chondrosarcomas, and rarely osteosarcomas [2]. The critical biomechanical importance of this weight bearing bone and the mimicking clinical features of various talus lesions warrant specific diagnosis for management. Lesions of talus usually present with chronic dull aching pain in the ankle associated with swelling and difficulty in weight bearing [3]. We present a case of nonspecific hindfoot pain in a patient having a talus lesion and concomitant varicose veins, synovitis of the ankle joint. The specific diagnosis of osteoblastoma of talus required a multidisciplinary approach which was managed with open curettage and bone grafting.

## Case presentation

**Figure 1:**
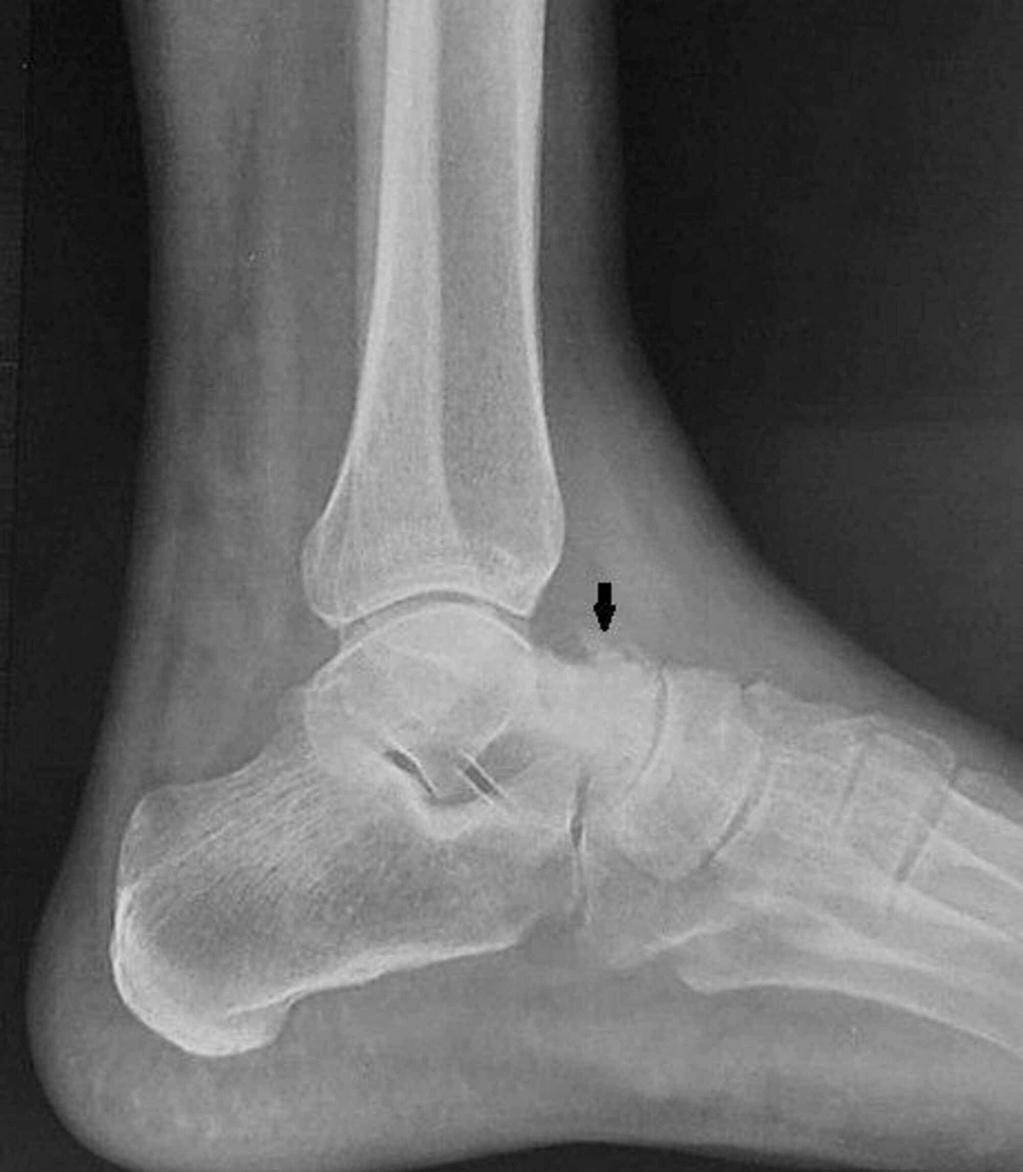
Lateral radiograph of the ankle and hindfoot demonstrates an exophytic sclerotic osseous lesion at neck of talus with punctate calcifications (black arrow)

A 45-year-old male patient presented to the orthopedics OPD with long standing painful swelling of the right ankle since 2 years. There was history of claudication in the right lower limb. There was no history of trauma or any other constitutional symptoms. The patient was a non-smoker and a teacher by profession. On clinical examination, there was diffused tenderness over right ankle and pain increased on passive dorsiflexion and plantar flexion of the foot. On visual analog scale, patient had a score of 8 on presentation. There was a bony hard swelling over the anterolateral aspect of ankle (over talus) with varicosities over the lateral aspect of leg. The patient had already taken multiple sclerosant injection for varicose veins. The hematological parameters were normal. Anteroposterior and lateral radiographs of the right ankle demonstrated an exophytic osseous lytic lesion with sclerotic margin and small foci of calcified matrix (Figure 1).

**Figure 2:**
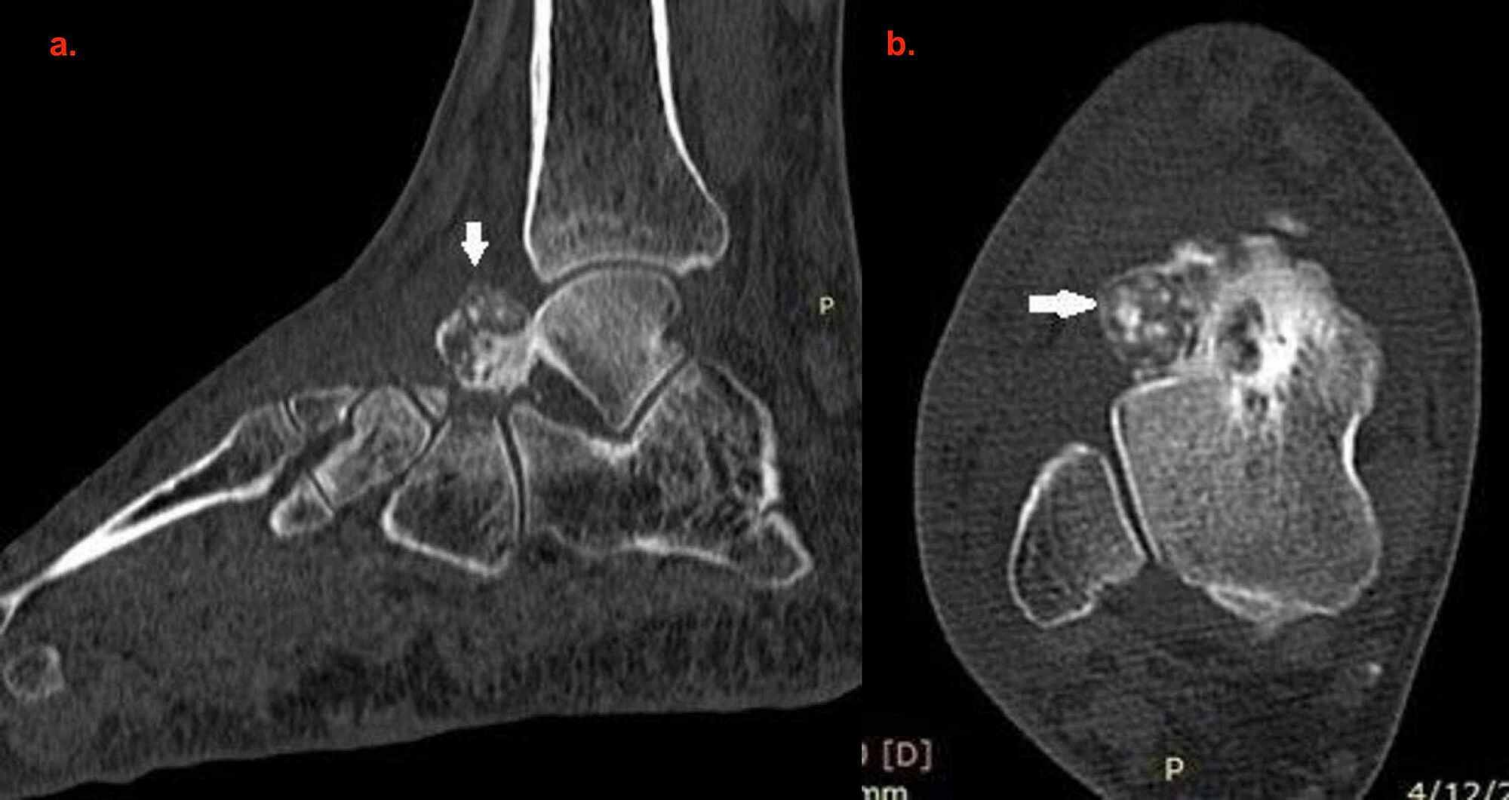
Sagittal reformatted (a) and axial (b) CT of the ankle and hindfoot demonstrates an exophytic capsulated osseous lesion at neck of talus having punctate calcifications (white arrow)

The zone of transition was normal (Figure 2).

**Figure 3:**
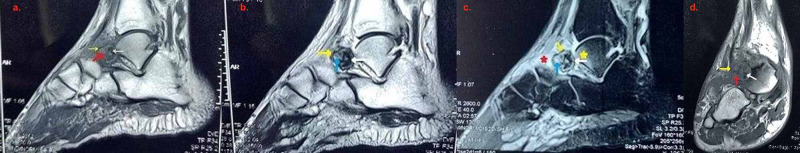
Sagittal T1 (a), T2 (b), STIR (c) and coronal T1 (d) MR images demonstrate an exophytic osseous lesion at neck of talus having thin hypointense capsule (yellow arrow). The matrix of the lesion demonstrates T1 isointense (to muscle) [Red arrow in fig. 3a, d] and T2 hyperintense (to muscle) [blue arrow in fig. 3b, c] with punctate signal voids (white arrow). The punctate signal voids corresponds to the calcifications as demonstrated in CT. Edema in adjacent marrow of talus (yellow asterisk) and in soft tissue of dorsum of foot (red asterisk) as demonstrated in fig. 3c.

Based on the radiographs, we had a differential diagnosis of osteoblastoma, chondroblastoma, and osteochondroma. To know the nature and extension of this lesion, an MRI was done which demonstrated tenosynovitis of tibialis anterior tendon, ankle joint effusion with an exophytic osseous lesion in talus and perilesional marrow edema (Figures 3A-3D).

 Further a Bone scan was planned which showed increased focal uptake in right talus. As patient also complained of claudication, a right lower limb arterial doppler was done, which demonstrated moderate stenotic changes in the distal anterior tibial artery and dorsalis pedis artery. 

To know the nature of tenosynovitis and ankle joint effusion, aspiration of ankle joint fluid was done which revealed serosanguinous fluid and was reported to be sterile. Synovial biopsy demonstrated only fibrocollagenous cells. 

**Figure 4:**
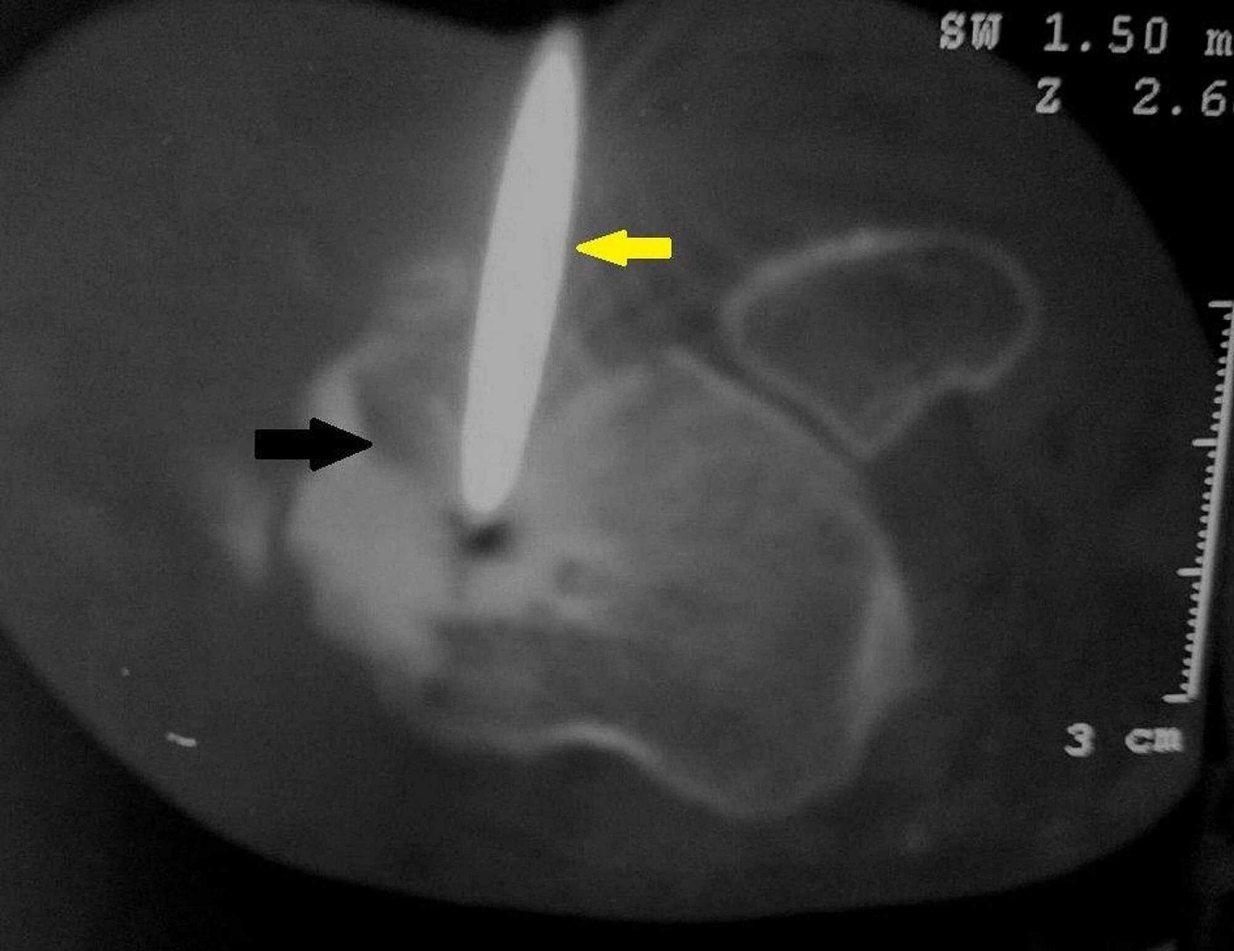
Axial CT of the hindfoot demonstrates CT guided biopsy of the talus neck lesion (black arrow) by the 11G bone biopsy needle (yellow arrow) through anterolateral approach

A CT-guided biopsy of the talus mass confirmed the diagnosis of osteoblastoma (Figure 4).

**Figure 5:**
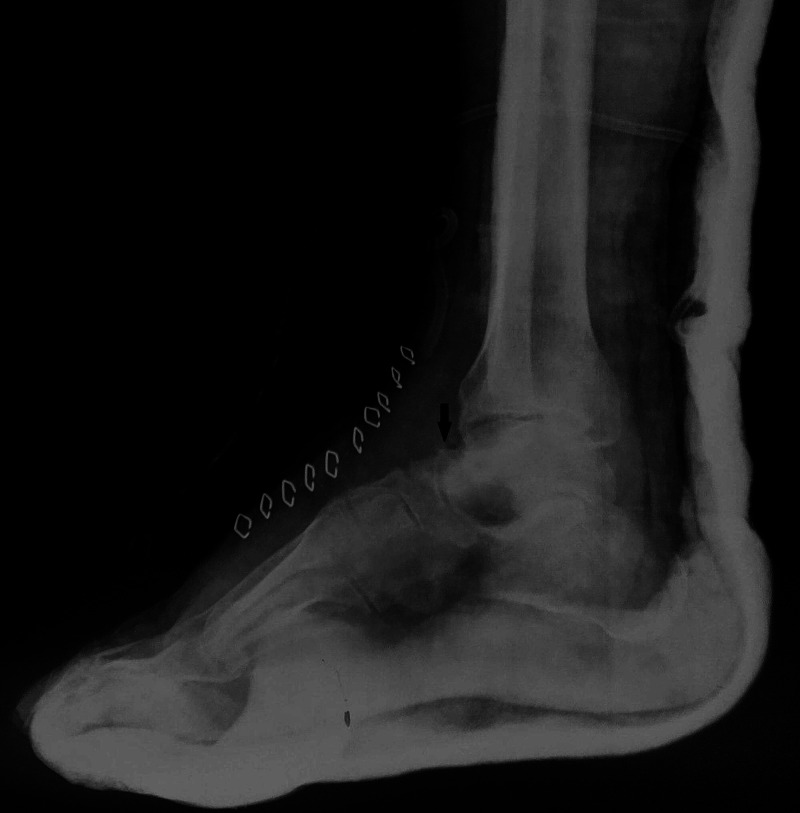
Post-operative lateral radiograph of the ankle and hindfoot demonstrates complete excision of the exophytic talus neck lesion (black arrow).

Surgical removal of the tumor was performed in supine position with a pneumatic tourniquet over the ipsilateral thigh. After preparation, sterile painting and draping, anterolateral approach to talus was used. Thorough soft tissue dissection was done and talus was exposed. An anterolateral exophytic growth was seen over the head of talus which was soft to probe as compared with the normal bone. No cortical collapse or fracture was seen. Tumor excision was done using a power burr and osteotome. Thorough extended curettage of the remaining lesion was done using a burr and hydrogen peroxide to achieve negative tumor margins. The cavity was irrigated with normal saline and packed with ipsilateral iliac crest bone graft. Closure of the wound was done and compression bandage applied. Post operatively, ankle was immobilized using a below knee posterior splint with ankle in neutral position for 6 weeks. Post-operative radiograph showed removal of the tumor with bone graft in the cavity (Figure 5).

## Discussion

Osteoblastoma is a rare benign tumor which forms an osteoid matrix, and accounts for less than one percent of all bone tumors [4]. It is predominantly seen in the 2nd and 3rd decade of life with males outnumbering females by a ratio 2:1 [5]. The common sites of involvement are the spine and long tubular bones. Osteoblastoma of the talus is considered a very rare condition and most commonly involves the neck of talus and involvement of body is reported to be rare [3].

Talar osteoblastomas usually present with chronic ankle pain, which is a localized, dull aching, and usually not nocturnal. Due to absence of response to salicylates, talus osteoblastoma may be wrongly diagnosed as ankle sprain [3]. Beytemur et al. presented a case of talar osteoblastoma which presented as type 1 complex regional pain syndrome [6]. However, our case patient presented with a nonspecific swelling of the ankle with inability to bear weight and multiple varicosities over lateral aspect of the leg with a history a claudication.

Temple et al. used plain radiographs, CT, MRI and a technitium bone scan to diagnose osteoblastoma [7]. However, in our case, since the lesion was masked by other pathologies such as varicose veins, ankle joint effusion and tenosynovitis, a multidisciplinary approach was taken and the patient underwent plain radiography, MRI, technitium bone scan and CT guided biopsy for the talar lesion, a synovial biopsy for tenosynovitis and doppler studies for claudication.

**Figure 6:**
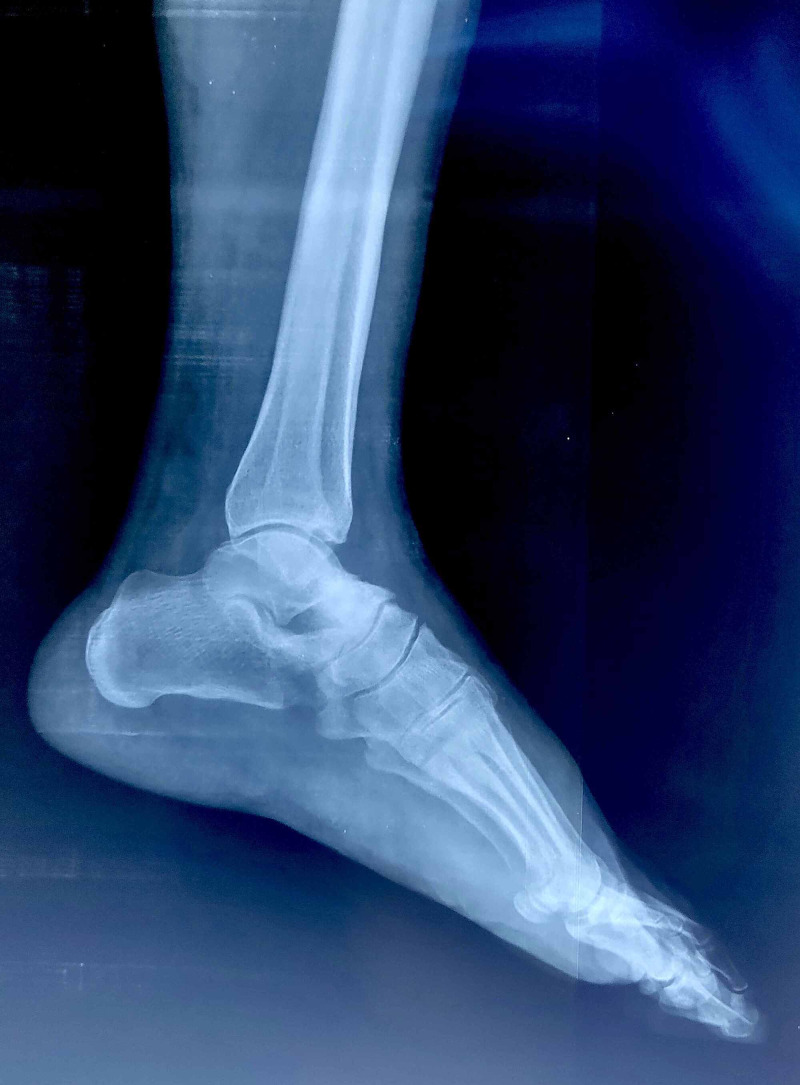
Lateral radiograph of the ankle and hindfoot 2 years post-operatively

Treatment options for osteoblastoma talus include open intralesional curettage with or without bone grafting, arthroscopic removal of the lesion and percutaneous radiofrequency ablation [3]. Itokazu et al. also reported ankle arthroplasty by subtotal talectomy for osteoblastomas of the talar body [8]. We decided to treat the lesion with conventional open curettage and bone grafting technique followed by immobilization of the ankle with a below knee splint for seven days with strict non-weight bearing. Pre-operatively on visual analog scale, score was 8 and immediate post operatively, score was 4. After seven days, below knee splint was removed and ankle was supported using a bulky dressing. Patient was advised passive ankle range of motion exercises with toe touch weight bearing using a height adjustable walker. After six weeks, patient was advised active ankle range of motion exercises with partial weight bearing. Full weight bearing was allowed after three months. After two years of follow-up patent has no pain in ankle with weight bearing and has full range of motion (Figure 6).

In our case, the patient had osteoblastoma of talus, the presentation of which was masked by pain due to tenosynovitis and varicose veins. Thus a multidisciplinary approach was required to hit the bull’s eye and appropriate treatment was started only after a thorough diagnostic workup.

## Conclusions

Osteoblastoma of the talus is a rare benign tumour occurring mainly in the second decade of life and presents with nonspecific clinical features around talus. It warrants a meticulous multidisciplinary diagnostic work-up for specific management, particularly when the talus lesion is associated with concomitant soft tissue and joint abnormalities.
